# Supplementary material for: Microglial depletion decreases Müller cell maturation and inner retinal vascular density
Source: Cell Commun Signal. 2025 Feb 17;23:90. doi: 10.1186/s12964-025-02083-5 (PMC11831819; doi:10.1186/s12964-025-02083-5)
Supplement: Supplementary file 3 — Supplementary Material 3 [file 12964_2025_2083_MOESM3_ESM.pdf]

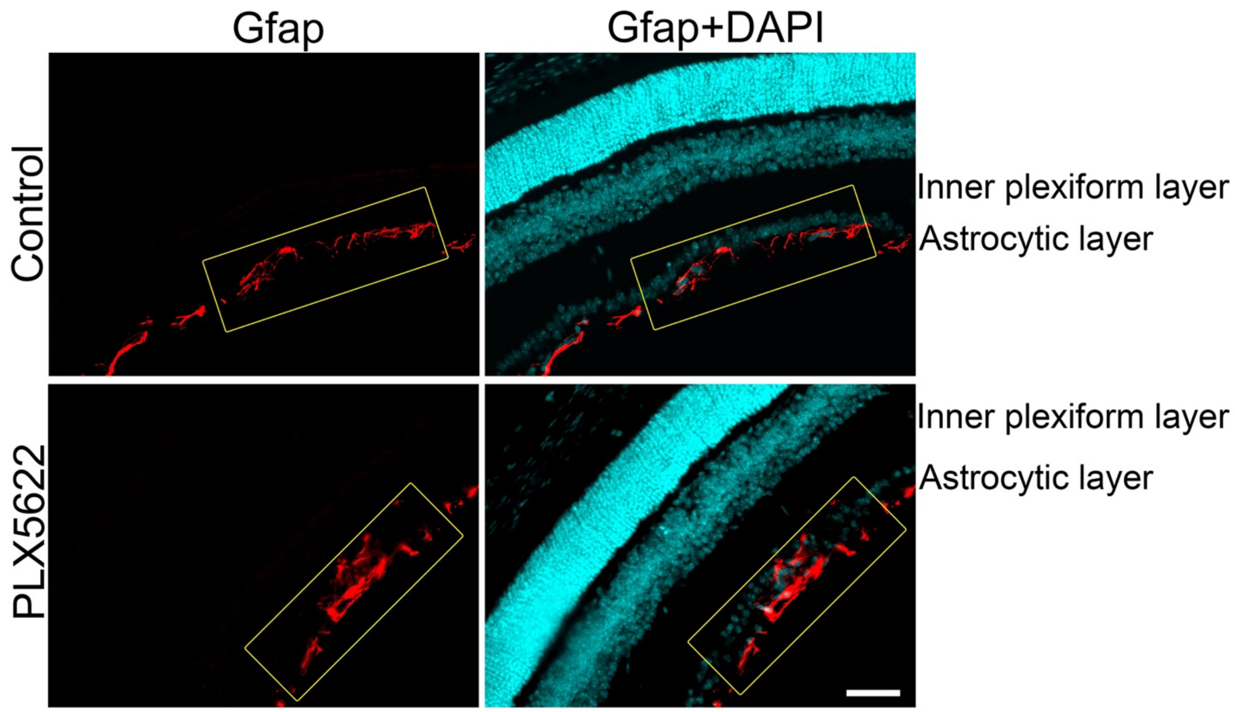


Figure S1: Gfap expression is restricted to astrocytic layer in the control and microglia depleted retinal sections. Representative P15 retinal sections from the control and PLX5622 groups (n=3) immunostained for Gfap and DAPI nuclear stain revealing Gfap expression only in the astrocytic layer (boxed area). Scale: 50 μm.

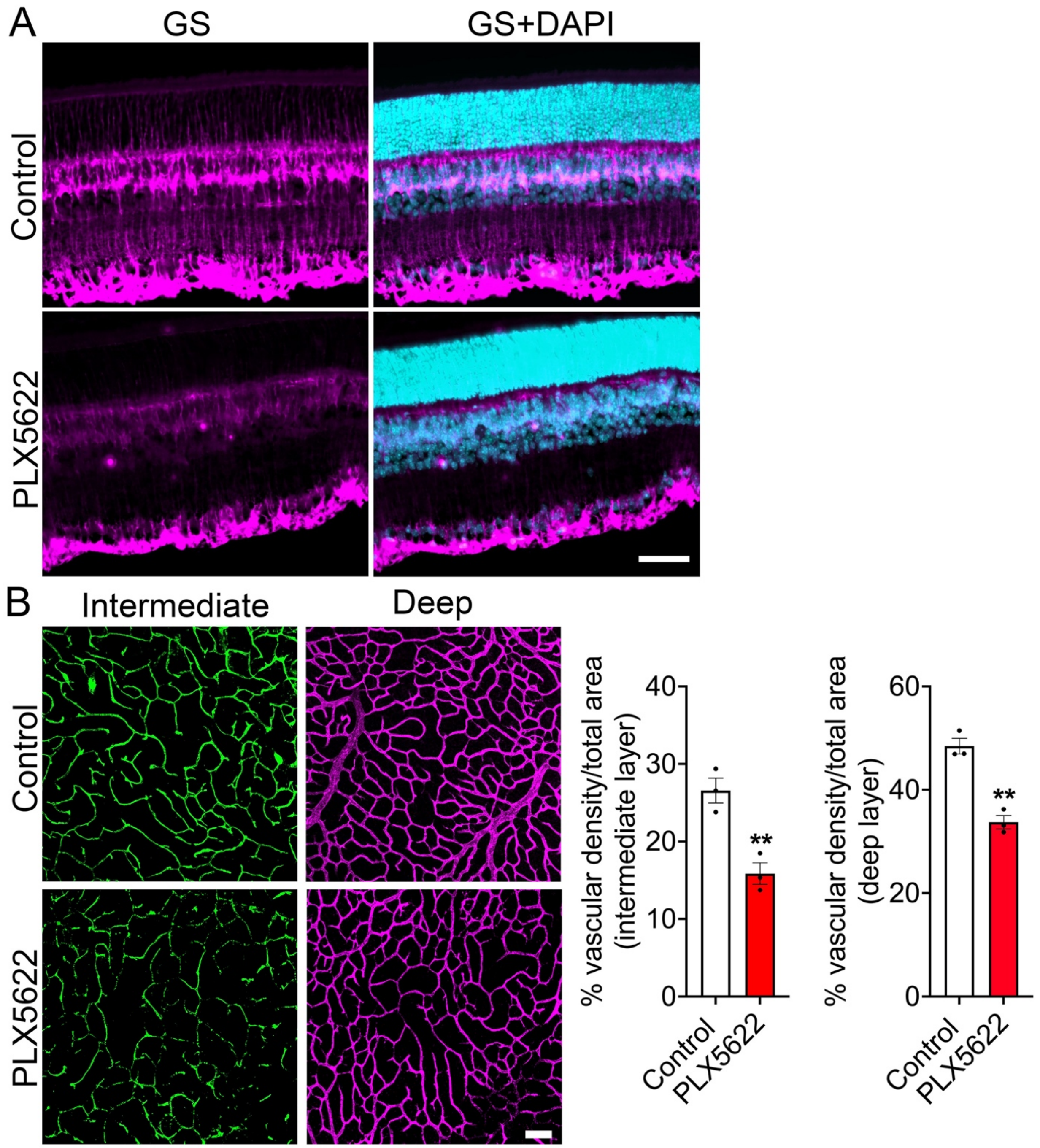


Figure S2. Microglia depletion resulted in reduced glutamine synthetase expression and inner retinal vascular density at P15. (A) Representative P15 retinal sections from the control and PLX5622 groups (n=3) immunostained for glutamine synthetase (GS) and DAPI nuclear stain to reveal GS expression in Müller cells. (B) P15 retinal flatmount immunostained for CD31 revealing intermediate and deep vascular layers in the control and PLX5622 groups (n=3). Bar graphs show the percentage of vascular density in the intermediate and deep vascular layers in the control and PLX5622 groups. Scale Bar: A and B are 50 μm.
